# Supplementary material for: Regional disparities in subjective wellbeing across Europe: A fuzzy hybrid TOPSIS approach
Source: PLoS One. 2026 Feb 9;21(2):e0341119. doi: 10.1371/journal.pone.0341119 (PMC12885272; doi:10.1371/journal.pone.0341119)
Supplement: S1 File — S1 Table. Regional subjective well-being rankings. This table reports the regional subjective well-being (R-SWB) scores and rankings for all NUTS2 and NUTS3 regions across the seven European countries included in the study, calculated using the Fuzzy-Hybrid TOPSIS approach. S2 Table. Quantile regression estimates. This table presents the estimated coefficients, standard errors, and significance levels of the quantile regression models at the 25th, 50th, and 75th percentiles of the subjective well-being distribution. (DOCX) [file pone.0341119.s001.docx]

Table S1: Regional Subjective well-being

| **Region** | **Country** | **R-SWB** | **Region** | **Country** | **R-SWB** |
| --- | --- | --- | --- | --- | --- |
| DOM - Overseas departments | FR | 1,000 | Cote-d'Or | FR | 0,552 |
| Saarland | DE | 0,878 | Lapland | FI | 0,552 |
| La Rioja | ES | 0,866 | Haut-Rhin | FR | 0,550 |
| Schleswig-Holstein | DE | 0,796 | Sarthe | FR | 0,550 |
| Aland | FI | 0,794 | Central Transdanubia | HU | 0,549 |
| Baleares | ES | 0,743 | Mayenne | FR | 0,548 |
| Bremen | DE | 0,724 | Alpes-Maritimes | FR | 0,548 |
| Navarra | ES | 0,715 | Tarn | FR | 0,547 |
| Bavaria | DE | 0,714 | Loir-et-Cher | FR | 0,547 |
| Mecklenburg-Vorpommern | DE | 0,711 | Orne | FR | 0,547 |
| Saxony | DE | 0,711 | Gironde | FR | 0,545 |
| Lot-et-Garonne | FR | 0,701 | Doubs | FR | 0,544 |
| North Rhine-Westphalia | DE | 0,701 | Marne | FR | 0,543 |
| Hautes-Alpes | FR | 0,701 | Haute-Garonne | FR | 0,542 |
| Allier | FR | 0,697 | Central Finland | FI | 0,541 |
| Baden-Wuerttemberg | DE | 0,697 | Varsinais-Suomi | FI | 0,541 |
| Cantabria | ES | 0,696 | Vaucluse | FR | 0,535 |
| Haute-Saone | FR | 0,694 | Eure-et-Loire | FR | 0,534 |
| Berlin-West | DE | 0,694 | Northern Hungary | HU | 0,534 |
| Berlin-East | DE | 0,682 | Kymenlaakso | FI | 0,532 |
| Koroska | SI | 0,678 | Finistere | FR | 0,531 |
| Rhineland-Palatinate | DE | 0,676 | Etelae-Savo | FI | 0,530 |
| Gorenjska | SI | 0,675 | Eure | FR | 0,529 |
| Thuringia | DE | 0,673 | Yvelines | FR | 0,524 |
| Hesse | DE | 0,673 | Ville de Paris | FR | 0,523 |
| Brandenburg | DE | 0,672 | Western Transdanubia | HU | 0,522 |
| Region Northern Jutland | DK | 0,666 | Savoie | FR | 0,522 |
| Meuse | FR | 0,665 | Seine-et-Marne | FR | 0,521 |
| Lower Saxony | DE | 0,662 | North Ostrobothnia | FI | 0,521 |
| Maine-et-Loire | FR | 0,661 | Bas-Rhin | FR | 0,520 |
| Central Region of Denmark | DK | 0,656 | Hautes-Pyrenees | FR | 0,520 |
| Hamburg | DE | 0,656 | Central Hungary | HU | 0,520 |
| Osrednja | SI | 0,655 | Ostrobothnia | FI | 0,517 |
| Saxony-Anhalt | DE | 0,650 | Uusimaa | FI | 0,516 |
| Pais Vasco | ES | 0,650 | Asturias | ES | 0,515 |
| Region de Murcia | ES | 0,647 | Vendee | FR | 0,515 |
| Capital Region of Denmark | DK | 0,646 | Meurthe-et-Moselle | FR | 0,514 |
| Dolenjska | SI | 0,642 | Can'tal | FR | 0,514 |
| Savinjska | SI | 0,640 | Isere | FR | 0,509 |
| Aveyron | FR | 0,636 | Charente | FR | 0,509 |
| Region Sealand | DK | 0,635 | Zasavska | SI | 0,509 |
| Pomurska | SI | 0,634 | Bouche-du-Rhone | FR | 0,508 |
| Cataluna | ES | 0,633 | Southern Great Plain | HU | 0,507 |
| Vosges | FR | 0,630 | North Karelia | FI | 0,504 |
| Castilla-Leon | ES | 0,629 | Hauts-de-Seine | FR | 0,500 |
| Morbihan | FR | 0,628 | Satakunta | FI | 0,499 |
| Gers | FR | 0,625 | Nievre | FR | 0,499 |
| Indre-et-Loire | FR | 0,623 | Obalnokraska | SI | 0,498 |
| Goriska | SI | 0,622 | Haute-Marne | FR | 0,494 |
| Dordogne | FR | 0,619 | Cher | FR | 0,494 |
| Extremadura | ES | 0,618 | Tarn-et-Garonne | FR | 0,491 |
| Region of Southern Denmark | DK | 0,618 | Var | FR | 0,487 |
| Deux-Sevres | FR | 0,617 | Yonne | FR | 0,484 |
| Charente-Maritime | FR | 0,614 | Creuse | FR | 0,484 |
| Central Ostrobothnia | FI | 0,614 | Pas-de-Calais | FR | 0,479 |
| Podravska | SI | 0,613 | Pohjois-Savo | FI | 0,472 |
| Paeijaet-Haeme | FI | 0,611 | Haute-Savoie | FR | 0,471 |
| Loire | FR | 0,610 | Northern Great Plain | HU | 0,464 |
| Aragon | ES | 0,607 | Pyrenees-Atlantiques | FR | 0,464 |
| Loiret | FR | 0,607 | Puy-de-Dome | FR | 0,463 |
| Comunidad Valenciana | ES | 0,604 | Seine-Saint-Denis | FR | 0,462 |
| Kainuu | FI | 0,602 | Calvados | FR | 0,461 |
| Lot | FR | 0,602 | Herault | FR | 0,460 |
| Jura | FR | 0,601 | Pyrenees-Orientales | FR | 0,455 |
| Corse | FR | 0,599 | Ardennes | FR | 0,453 |
| Saone-et-Loire | FR | 0,595 | Ardeche | FR | 0,452 |
| Drome | FR | 0,594 | South Karelia | FI | 0,451 |
| Andalucia | ES | 0,591 | Cotes-d'Armor | FR | 0,448 |
| Aude | FR | 0,591 | Oise | FR | 0,445 |
| Castilla-La Mancha | ES | 0,591 | Haute-Vienne | FR | 0,428 |
| Nord | FR | 0,582 | Ain | FR | 0,428 |
| Madrid | ES | 0,579 | South Ostrobothnia | FI | 0,427 |
| Pirkanmaa | FI | 0,578 | Correze | FR | 0,419 |
| Galicia | ES | 0,575 | Southern Transdanubia | HU | 0,413 |
| Gard | FR | 0,573 | Ariege | FR | 0,400 |
| Alpes-de-Haute-Provence | FR | 0,570 | Vienne | FR | 0,397 |
| Budapest | HU | 0,570 | Moselle | FR | 0,394 |
| Kraska | SI | 0,568 | Aisne | FR | 0,385 |
| Seine-Maritime | FR | 0,565 | Belfort (Territoire) | FR | 0,382 |
| Manche | FR | 0,561 | Somme | FR | 0,378 |
| Rhone | FR | 0,560 | Ceuta | ES | 0,377 |
| Essone | FR | 0,558 | Val-de-Marne | FR | 0,368 |
| Haute-Loire | FR | 0,557 | Aube | FR | 0,361 |
| Canarias | ES | 0,557 | Landes | FR | 0,347 |
| Spodnjeposavska | SI | 0,556 | Val-d'Oise | FR | 0,344 |
| Kanta-Haeme | FI | 0,555 | Indre | FR | 0,316 |
| Loire-Atlantique | FR | 0,554 | Itae-Uusimaa | FI | 0,200 |
| Ille-et-Vilaine | FR | 0,553 |  |  |  |
| Own elaboration; R-SWB: Regional Subjective well-being; DK: Denmark, FR: France; ES: Spain; DE: Germany; FI: Finland; HU: Hungary; SI: Slovenia | | | | | |

Table S2: Quantile regression

| Subjective well-being | Coef. | Std. error | Sig. | Coef. | Std. error | Sig. | Coef. | Std. error | Sig. |
| --- | --- | --- | --- | --- | --- | --- | --- | --- | --- |
|  | 25^th^ quantile  Pseudo-R^2^: 0.480 | | | Median quantile  Pseudo-R^2^: 0.393 | | | 75^th^ quantile  Pseudo-R^2^: 0.447 | | |
| Country (Spain = 0) | | | | | | | | | |
| Denmark | 0,02 | 0,010 |  | 0,01 | 0,009 |  | 0,00 | 0,009 |  |
| Finland | -0,05 | 0,009 | *** | -0,06 | 0,008 | *** | -0,06 | 0,009 | *** |
| France | -0,03 | 0,007 | *** | -0,04 | 0,006 | *** | -0,05 | 0,007 | *** |
| Germany | 0,06 | 0,008 | *** | 0,04 | 0,007 | *** | 0,02 | 0,008 | ** |
| Hungary | -0,07 | 0,008 | *** | -0,07 | 0,007 | *** | -0,07 | 0,008 | *** |
| Slovenia | 0,03 | 0,008 | *** | 0,00 | 0,007 |  | -0,02 | 0,008 | * |
| Age (under 24 = 0) | | | | | | | | | |
| 25-35 | 0,03 | 0,010 | *** | 0,05 | 0,008 | *** | 0,04 | 0,009 | *** |
| 36-45 | 0,05 | 0,009 | *** | 0,06 | 0,008 | *** | 0,06 | 0,009 | *** |
| 46-55 | 0,07 | 0,009 | *** | 0,07 | 0,008 | *** | 0,06 | 0,009 | *** |
| 56-65 | 0,10 | 0,009 | *** | 0,09 | 0,008 | *** | 0,09 | 0,009 | *** |
| 66-75 | 0,12 | 0,010 | *** | 0,12 | 0,009 | *** | 0,10 | 0,009 | *** |
| over 75 | 0,12 | 0,012 | *** | 0,13 | 0,010 | *** | 0,11 | 0,011 | *** |
| Age NA | 0,11 | 0,029 | *** | 0,05 | 0,025 | * | 0,05 | 0,027 |  |
| Gender (Male = 0) | | | | | | | | |  |
| Female | -0,02 | 0,004 | *** | -0,02 | 0,004 | *** | -0,01 | 0,004 | *** |
| Education (Upper lever tertiary = 0) | | | | | | | | | |
| No formal Education | 0,01 | 0,016 |  | -0,01 | 0,014 |  | 0,01 | 0,015 |  |
| Primary school | 0,02 | 0,011 |  | 0,02 | 0,009 | * | 0,03 | 0,010 | *** |
| Lower secondary | 0,00 | 0,008 |  | 0,01 | 0,007 |  | 0,02 | 0,007 | ** |
| Upper secondary | 0,02 | 0,008 | * | 0,01 | 0,007 | * | 0,02 | 0,007 | * |
| Post-secondary | 0,02 | 0,008 | *** | 0,02 | 0,007 | * | 0,02 | 0,007 | *** |
| Lower level tertiary | 0,01 | 0,007 |  | 0,01 | 0,006 | * | 0,02 | 0,007 | *** |
| Education NA | 0,09 | 0,031 | *** | 0,07 | 0,026 | ** | 0,00 | 0,028 |  |
| Religion (Catholic = 0) | | | | | | | | | |
| No religion | -0,01 | 0,006 |  | -0,01 | 0,005 | * | 0,00 | 0,005 |  |
| Protestant | -0,01 | 0,007 |  | -0,01 | 0,006 | * | -0,01 | 0,007 | * |
| Orthodox | 0,00 | 0,019 |  | -0,01 | 0,016 |  | 0,00 | 0,018 |  |
| Other-Christian | -0,02 | 0,029 |  | -0,01 | 0,025 |  | -0,01 | 0,027 |  |
| Jewish | -0,09 | 0,052 |  | -0,11 | 0,045 | * | -0,06 | 0,049 |  |
| Buddhist | -0,03 | 0,017 | * | 0,00 | 0,015 |  | 0,00 | 0,016 |  |
| Hindu | -0,08 | 0,049 |  | -0,02 | 0,042 |  | -0,04 | 0,045 |  |
| Other Asian Religions | -0,03 | 0,031 |  | 0,00 | 0,027 |  | -0,03 | 0,029 |  |
| Other religions | -0,02 | 0,017 |  | -0,04 | 0,015 | * | -0,04 | 0,016 | ** |
| Attend (Several times a week = 0) | | | | | | | | | |
| Once a week | -0,02 | 0,020 |  | 0,02 | 0,018 |  | 0,01 | 0,019 |  |
| 2 or 3 times a month | -0,03 | 0,022 |  | 0,01 | 0,019 |  | 0,01 | 0,020 |  |
| Once a month | -0,01 | 0,022 |  | 0,03 | 0,019 |  | 0,01 | 0,021 |  |
| Several times a year | -0,02 | 0,019 |  | 0,02 | 0,017 |  | 0,00 | 0,018 |  |
| Once a year | -0,02 | 0,020 |  | 0,02 | 0,017 |  | 0,01 | 0,019 |  |
| less frequently than once a year | -0,02 | 0,019 |  | 0,01 | 0,017 |  | 0,00 | 0,018 |  |
| never | -0,02 | 0,019 |  | 0,02 | 0,017 |  | 0,01 | 0,018 |  |
| attend NA | 0,00 | 0,026 |  | 0,03 | 0,023 |  | 0,01 | 0,024 |  |
| Income (Highest = 0) | | | | | | | | | |
| Lowest | -0,17 | 0,031 | *** | -0,15 | 0,026 |  | -0,11 | 0,028 | *** |
| income2 | -0,15 | 0,029 | *** | -0,14 | 0,025 |  | -0,12 | 0,027 | *** |
| income3 | -0,12 | 0,027 | *** | -0,11 | 0,024 |  | -0,09 | 0,025 | *** |
| income4 | -0,08 | 0,027 | ** | -0,09 | 0,023 |  | -0,07 | 0,025 | *** |
| income5 | -0,04 | 0,026 |  | -0,05 | 0,023 |  | -0,04 | 0,024 |  |
| income6 | -0,03 | 0,026 |  | -0,04 | 0,023 |  | -0,03 | 0,024 |  |
| income7 | -0,01 | 0,026 |  | -0,03 | 0,023 |  | -0,02 | 0,024 |  |
| income8 | 0,00 | 0,027 |  | 0,00 | 0,023 |  | 0,00 | 0,025 |  |
| income9 | 0,03 | 0,030 |  | 0,00 | 0,026 |  | 0,01 | 0,028 |  |
| Income NA | -0,07 | 0,028 | * | -0,05 | 0,024 |  | -0,04 | 0,026 |  |
| Health status (Excellent = 0) | | | | | | | | | |
| Poor | -0,29 | 0,012 | *** | -0,27 | 0,011 |  | -0,25 | 0,011 | *** |
| Fair | -0,18 | 0,009 | *** | -0,18 | 0,007 |  | -0,17 | 0,008 | *** |
| Good | -0,09 | 0,007 | *** | -0,09 | 0,006 |  | -0,11 | 0,007 | *** |
| Very good | -0,03 | 0,008 | *** | -0,04 | 0,007 |  | -0,06 | 0,007 | *** |
| Health NA | -0,10 | 0,026 | *** | -0,11 | 0,023 |  | -0,11 | 0,024 | *** |
| _cons | 0,74 | 0,034 | *** | 0,81 | 0,029 |  | 0,91 | 0,031 | *** |
| ***: p value < 0.001; **: p value < 0.01; *: p value < 0.05. | | | | | | | | | |
