# Supplementary material for: Regional disparities in subjective wellbeing across Europe: A fuzzy hybrid TOPSIS approach
Source: PLoS One. 2026 Feb 9;21(2):e0341119. doi: 10.1371/journal.pone.0341119 (PMC12885272; doi:10.1371/journal.pone.0341119)
Supplement: Attachment — Submitted filename: comments 8 May (1).docx [file pone.0341119.s002.docx]

Thank you for giving me the opportunity to review the manuscript titled “Regional Disparities in Subjective Wellbeing Across Europe: A Fuzzy Hybrid TOPSIS Approach”. Although the manuscript is written very well but there are some changes that need to be made in manuscript.

1. Abstract part needs to be revised; it would be better if numerical data is stated along with the claims which author makes for providing better information to the readers.
2. Author can consider splitting long sections into smaller sub sections for clarity especially methodology and results so that it becomes easier for reader to understand the context.
3. Fuzzy Hybrid approach methodology has been used to create synthetic indicator but the paper lacks proper theoretical framing that justify why this method has been used beyond “questionnaire data is vague”. Some more arguments need to be given that justify the use of this method instead of some other traditional methods.
4. Discussions on why capital of different regions underperformed except Berlin needs to be explained more.
5. Justification needs to be given why 25^th^, Median and 75^th^ quintile is being taken. Authors need to highlight more studies that justify the reason behind choosing this distribution symmetry.
6. Spatial plotting, graphical representation can be given instead of clustered tables to make it more easily understandable.
7. In order to ensure the precision of the results confidence interval needs to be given along- side the coefficient.
8. Although, the idea of the study is noble and the objectives are interesting, and overall manuscript is fine but the authors need to put more emphasis on the policy implication. The way in which it can help in coping regional disparities in subjective well- being across Europe.
