# Supplementary material for: Regional disparities in subjective wellbeing across Europe: A fuzzy hybrid TOPSIS approach
Source: PLoS One. 2026 Feb 9;21(2):e0341119. doi: 10.1371/journal.pone.0341119 (PMC12885272; doi:10.1371/journal.pone.0341119)
Supplement: Attachment — Submitted filename: Response to Reviewers.docx [file pone.0341119.s003.docx]

**Manuscript Number: PONE-D-25-21410**

**Title: Regional Disparities in Subjective Wellbeing Across Europe: A Fuzzy Hybrid TOPSIS Approach**

The authors acknowledge the editor and reviewers for their comments that improved the paper's presentation. This document outlines our responses to the editor and reviewers’ comments. Our responses are italicized and indicated by a “🡪” sign.

# Editor

28-Nov-2025

Dear,

Thank you for submitting your manuscript to PLOS ONE. After careful consideration, we feel that it has merit but does not fully meet PLOS ONE’s publication criteria as it currently stands. Therefore, we invite you to submit a revised version of the manuscript that addresses the points raised during the review process.

Please submit your revised manuscript by Jan 12 2026 11:59PM. If you will need more time than this to complete your revisions, please reply to this message or contact the journal office at [plosone@plos.org](mailto:plosone@plos.org). When you're ready to submit your revision, log on to https://www.editorialmanager.com/pone/ and select the 'Submissions Needing Revision' folder to locate your manuscript file.

Please include the following items when submitting your revised manuscript:

- A rebuttal letter that responds to each point raised by the academic editor and reviewer(s). You should upload this letter as a separate file labeled 'Response to Reviewers'.
- A marked-up copy of your manuscript that highlights changes made to the original version. You should upload this as a separate file labeled 'Revised Manuscript with Track Changes'.
- An unmarked version of your revised paper without tracked changes. You should upload this as a separate file labeled 'Manuscript'.

If you would like to make changes to your financial disclosure, please include your updated statement in your cover letter. Guidelines for resubmitting your figure files are available below the reviewer comments at the end of this letter.

If applicable, we recommend that you deposit your laboratory protocols in protocols.io to enhance the reproducibility of your results. Protocols.io assigns your protocol its own identifier (DOI) so that it can be cited independently in the future. For instructions see: [https://journals.plos.org/plosone/s/submission-guidelines#loc-laboratory-protocols](https://track.editorialmanager.com/CL0/https:%2F%2Fjournals.plos.org%2Fplosone%2Fs%2Fsubmission-guidelines%23loc-laboratory-protocols/1/010f019ac96bb736-0d488736-c7e3-422f-88c7-eaecdea4d0c7-000000/d7AjzN0e7NUzxh-nh-s0vxdKzYkMVqPMHgS6iGgjndA=239). Additionally, PLOS ONE offers an option for publishing peer-reviewed Lab Protocol articles, which describe protocols hosted on protocols.io. Read more information on sharing protocols at [https://plos.org/protocols?utm_medium=editorial-email&utm_source=authorletters&utm_campaign=protocols](https://track.editorialmanager.com/CL0/https:%2F%2Fplos.org%2Fprotocols%3Futm_medium=editorial-email%26utm_source=authorletters%26utm_campaign=protocols/1/010f019ac96bb736-0d488736-c7e3-422f-88c7-eaecdea4d0c7-000000/V5ZDgf9WWIaXarDYeoyAwGsr3rMnryyGYwFmydG5gAU=239).

We look forward to receiving your revised manuscript.

Kind regards,

Shaonong Dang, PhD

Academic Editor

PLOS ONE

# Editor and Reviewer comments

Journal Requirements:

When submitting your revision, we need you to address these additional requirements.

1. Please ensure that your manuscript meets PLOS ONE's style requirements, including those for file naming. The PLOS ONE style templates can be found at

https://journals.plos.org/plosone/s/file?id=wjVg/PLOSOne_formatting_sample_main_body.pdf and

https://journals.plos.org/plosone/s/file?id=ba62/PLOSOne_formatting_sample_title_authors_affiliations.pdf

 🡪 Now, the manuscript meets the PLOS ONE's style requirements

2. We suggest you thoroughly copyedit your manuscript for language usage, spelling, and grammar. If you do not know anyone who can help you do this, you may wish to consider employing a professional scientific editing service.

 🡪 We have checked the manuscript with a native English speaker.

The American Journal Experts (AJE) (https://www.aje.com/) is one such service that has extensive experience helping authors meet PLOS guidelines and can provide language editing, translation, manuscript formatting, and figure formatting to ensure your manuscript meets our submission guidelines. Please note that having the manuscript copyedited by AJE or any other editing services does not guarantee selection for peer review or acceptance for publication.

Upon resubmission, please provide the following:

- The name of the colleague or the details of the professional service that edited your manuscript
- A copy of your manuscript showing your changes by either highlighting them or using track changes (uploaded as a *supporting information* file)
- A clean copy of the edited manuscript (uploaded as the new *manuscript* file)

🡪 We have solved this issue.

3. We note that the grant information you provided in the ‘Funding Information’ and ‘Financial Disclosure’ sections do not match.

When you resubmit, please ensure that you provide the correct grant numbers for the awards you received for your study in the ‘Funding Information’ section.

🡪 We have solved this issue.

4. Thank you for stating the following financial disclosure:

“*personal information*”

Please state what role the funders took in the study.  If the funders had no role, please state: "The funders had no role in study design, data collection and analysis, decision to publish, or preparation of the manuscript." If this statement is not correct you must amend it as needed.

🡪 We have solved this issue.

Please include this amended Role of Funder statement in your cover letter; we will change the online submission form on your behalf.

🡪 We have solved this issue.

5. Please note that funding information should not appear in the Acknowledgments section or other areas of your manuscript. We will only publish funding information present in the Funding Statement section of the online submission form. Please remove any funding-related text from the manuscript.

🡪 We have solved this issue.

6. We noted in your submission details that a portion of your manuscript may have been presented or published elsewhere:

 “Data openly available in a public repository that issues datasets with DOI doi.org/10.4232/1.13322”

 Please clarify whether this [conference proceeding or publication] was peer-reviewed and formally published. If this work was previously peer-reviewed and published, in the cover letter please provide the reason that this work does not constitute dual publication and should be included in the current manuscript.

🡪 We have now specified the availability of that as follows: All data underlying the findings are openly available in the ISSP Data Archive at GESIS – Leibniz Institute for the Social Sciences, with DOI doi.org/10.4232/1.13322, and have not been used to publish any previous analysis overlapping with the present manuscript.

7. Please note that your Data Availability Statement is currently missing the repository name. If your manuscript is accepted for publication, you will be asked to provide these details on a very short timeline. We therefore suggest that you provide this information now, though we will not hold up the peer review process if you are unable.

🡪 We have solved this issue.

8. We note you have included a table to which you do not refer in the text of your manuscript. Please ensure that you refer to Tables 6 and 7 in your text; if accepted, production will need this reference to link the reader to the Table.

🡪 We have solved this issue.

9. If the reviewer comments include a recommendation to cite specific previously published works, please review and evaluate these publications to determine whether they are relevant and should be cited. There is no requirement to cite these works unless the editor has indicated otherwise.

🡪 Thank you for your comments. We hope that the new version of the manuscript can be accepted for publication.

**Additional Editor Comments:**

It is an interesting study where authors investigated the regional disparities in Subjective Wellbeing across Europe. The reviewers have raised some concerns, and authors are suggested to address them carefully to further improve the manuscript.


[Note: HTML markup is below. Please do not edit.]

# Reviewer comments

Reviewer's Responses to Questions

**Comments to the Author**

1. Is the manuscript technically sound, and do the data support the conclusions?

The manuscript must describe a technically sound piece of scientific research with data that supports the conclusions. Experiments must have been conducted rigorously, with appropriate controls, replication, and sample sizes. The conclusions must be drawn appropriately based on the data presented.

Reviewer #1: Yes

Reviewer #2: Yes

2. Has the statistical analysis been performed appropriately and rigorously?

Reviewer #1: Yes

Reviewer #2: No

3. Have the authors made all data underlying the findings in their manuscript fully available?

The [PLOS Data policy](http://track.editorialmanager.com/CL0/http:%2F%2Fwww.plosone.org%2Fstatic%2Fpolicies.action%23sharing/1/010f019ac96bb736-0d488736-c7e3-422f-88c7-eaecdea4d0c7-000000/ukqPIqQzvcCZznOwUGolURfJ4u7LAIsx6DohR9v30Pc=239) requires authors to make all data underlying the findings described in their manuscript fully available without restriction, with rare exception (please refer to the Data Availability Statement in the manuscript PDF file). The data should be provided as part of the manuscript or its supporting information, or deposited to a public repository. For example, in addition to summary statistics, the data points behind means, medians and variance measures should be available. If there are restrictions on publicly sharing data—e.g. participant privacy or use of data from a third party—those must be specified.

Reviewer #1: No

Reviewer #2: Yes

4. Is the manuscript presented in an intelligible fashion and written in standard English?

PLOS ONE does not copyedit accepted manuscripts, so the language in submitted articles must be clear, correct, and unambiguous. Any typographical or grammatical errors should be corrected at revision, so please note any specific errors here.

Reviewer #1: Yes

Reviewer #2: Yes

5. Review Comments to the Author

Please use the space provided to explain your answers to the questions above. You may also include additional comments for the author, including concerns about dual publication, research ethics, or publication ethics. (Please upload your review as an attachment if it exceeds 20,000 characters)

Reviewer #1: the idea of the study is noble and the objectives are interesting, and overall manuscript is fine but the authors need to address cerain issues like providing proper justification behind choosig a method and why ceratin indicators are being used

🡪 Thank you for your comments. We hope that the new version of the manuscript can be accepted for publication.

Reviewer #2: Thank you for the invitation to review manuscript titled “Regional Disparities in Subjective Wellbeing Across Europe: A Fuzzy Hybrid TOPSIS Approach”. I believe that this manuscript is interesting. However, there are areas in which this manuscript must be improved before it would be suitable for publication. The following is a list of points necessary to improve the paper and which justify the decision to Major Revision.

- The introduction is too brief. The paragraph in the data section justifying the countries analysed should be in the introduction. Furthermore, the data is eight years old, so it should be justified that this time lapse is not relevant to the results obtained. For example, by demonstrating that the World Happiness Index between 2017 and 2025 has not changed significantly.

🡪 We thank the reviewer for this comment. The introduction has been expanded accordingly. Firstly, the justification for selecting the seven countries has been added to the Introduction and rewritten to clarify the goal of ensuring broad territorial representation across EU regions. Secondly, we have added a brief explanation to demonstrate that the eight-year gap does not affect the validity of the findings. The World Happiness Report rankings for these countries remained largely stable between 2017 and 2024, indicating no significant changes in relative well-being levels. These additions strengthen the study's contextualisation and justify the data's temporal relevance.

- The Theoretical background section is not up to date; articles from the last five years are not mentioned. It should be expanded with more recent literature on the context of the research.

🡪 We appreciate this helpful observation. In the revised manuscript, the “Brief Theoretical Background” section has been substantially updated and expanded to incorporate literature from the last five years. Specifically, we now discuss:

i. new multidimensional approaches to SWB;
ii. evidence on regional disparities in SWB across Europe;
iii. updated findings on the role of socioeconomic factors, such as age, education, income and wealth.

These additions ensure that the theoretical framework reflects current research and situates our contribution more effectively within the recent literature. All changes are visible in the revised Theoretical Background section.

- The title of Table 1 is incorrect because it does not include the maximum, minimum, mean, and standard deviation statistics. The title should be changed. And for the seven countries, a table with the aforementioned statistics should be included.

🡪 The title of Table 1 has been corrected to reflect its content as “Sample Composition by Country and Socio-Demographic Characteristics” instead of “Descriptive statistics.”

- It is stated that data is available at NUTS2 and NUTS3 levels, but it is not explained whether the mixed granularity affects comparability (e.g. France NUTS3 vs other countries NUTS2). This should be clarified.

🡪 We thank the reviewer for this important observation. In the revised manuscript, we have added an explanation to clarify why the mixed territorial granularity (NUTS2 for most countries and NUTS3 for France) does not affect the comparability of our analysis. Specifically, the Fuzzy-Hybrid TOPSIS indicator relies on the relative distances between defuzzified values, rather than absolute regional size or administrative scale. Consequently, the method independently evaluates each territorial unit, regardless of whether it corresponds to a NUTS2 or NUTS3 region.

- Elasticities are not standard in TOPSIS; it would be advisable to justify the method better or provide more detailed references.

🡪 We appreciate the reviewer’s comment. In the revised manuscript, we have expanded the methodological explanation of the elasticity measure and added supporting references. Although elasticities are not part of the traditional TOPSIS framework, they have been introduced in recent studies to assess the marginal influence of each item on the synthetic indicator. Following Martín & Indelicato (2022) and other applications in fuzzy multi-criteria decision-making (MCDM), elasticities provide an interpretable sensitivity measure that complements the TOPSIS score without altering the method’s core structure. We have clarified this rationale and added detailed references and explanations in the Methodology section.

- Quantile regression is applied to a continuous indicator derived from TOPSIS, but the covariates have potentially relevant correlations (age, education, health, income). A correlation matrix (or at least VIF for multicollinearity) must be included in the text.

🡪 We appreciate this comment. In the revised manuscript, we now include multicollinearity diagnostics to assess the correlations among age, education, health, and other covariates. Specifically, we show that all VIF values fall well below commonly accepted thresholds (maximum VIF = 3.19). This confirms that multicollinearity is not a concern in our quantile regression specification.

- Table 7 should include the Pseudo-R² by quantile
  Robustness techniques for estimation must be specified.

🡪 Table A1 has been updated to include the Pseudo-R² values for each estimated quantile (0.25, 0.50 and 0.75).

- The conclusions should not include statements such as: The results agree with other studies (Smith & Diekmann, 2017; Tovar & Bourdeau-Lepage, 2013; Churchill et al., 2019; Cohen, 2002; Steptoe et al., 2015; Thomson et al., 2022; Varela et al., 2021). This should be included in the discussion. The conclusions should highlight the main contribution of the research, its limitations and future lines of research.

🡪 We thank the reviewer for this observation. We have revised the Conclusions section to remove statements comparing our results with prior studies and moved them to the Discussion section.

6. PLOS authors have the option to publish the peer review history of their article ([what does this mean?](https://track.editorialmanager.com/CL0/https:%2F%2Fjournals.plos.org%2Fplosone%2Fs%2Feditorial-and-peer-review-process%23loc-peer-review-history/1/010f019ac96bb736-0d488736-c7e3-422f-88c7-eaecdea4d0c7-000000/CRFJs_foZ_idHUrtGAAfrsjbiuTF3w5zxxl2K7DC9Ko=239)). If published, this will include your full peer review and any attached files.


If you choose “no”, your identity will remain anonymous but your review may still be made public.


**Do you want your identity to be public for this peer review?** For information about this choice, including consent withdrawal, please see our [Privacy Policy](https://track.editorialmanager.com/CL0/https:%2F%2Fwww.plos.org%2Fprivacy-policy/1/010f019ac96bb736-0d488736-c7e3-422f-88c7-eaecdea4d0c7-000000/HHiSOFfRj9kDBNRDB86Mtv3G4HJTSHmFO3XNo0aV2_8=239).

Reviewer #1: No

Reviewer #2: No

[NOTE: If reviewer comments were submitted as an attachment file, they will be attached to this email and accessible via the submission site. Please log into your account, locate the manuscript record, and check for the action link "View Attachments". If this link does not appear, there are no attachment files.]

To ensure your figures meet our technical requirements, please review our figure guidelines: <https://journals.plos.org/plosone/s/figures>

You may also use PLOS’s free figure tool, NAAS, to help you prepare publication quality figures: <https://journals.plos.org/plosone/s/figures#loc-tools-for-figure-preparation>.

NAAS will assess whether your figures meet our technical requirements by comparing each figure against our figure specifications.

Thank you for giving me the opportunity to review the manuscript titled “Regional Disparities in Subjective Wellbeing Across Europe: A Fuzzy Hybrid TOPSIS Approach”. Although the manuscript is written very well but there are some changes that need to be made in manuscript.

1. Abstract part needs to be revised; it would be better if numerical data is stated along with the claims which author makes for providing better information to the readers.

🡪 The abstract has been revised to include key numerical results, providing readers with a more precise and more informative overview.

2. Author can consider splitting long sections into smaller sub sections for clarity especially methodology and results so that it becomes easier for reader to understand the context.

🡪 We appreciate the reviewer’s observation regarding the length of the Methodology and Results sections. In the revised manuscript, these sections have been reorganised into clearly marked subsections, improving readability and facilitating a more structured presentation of the analytical process and findings.

3. Fuzzy Hybrid approach methodology has been used to create synthetic indicator but the paper lacks proper theoretical framing that justify why this method has been used beyond “questionnaire data is vague”. Some more arguments need to be given that justify the use of this method instead of some other traditional methods.

🡪 We thank the reviewer for this insightful comment. In the revised manuscript, we have substantially strengthened the theoretical justification for employing the Fuzzy-Hybrid TOPSIS (FHT) methodology. We now clarify why this approach is particularly appropriate and why traditional statistical methods are insufficient for this type of measurement.

4. Discussions on why capital of different regions underperformed except Berlin needs to be explained more.

🡪 We appreciate this valuable comment. In the revised manuscript, the discussion has been expanded to explain more clearly why the capitals of different regions underperformed except Berlin.

5. Justification needs to be given why 25^th^, Median and 75^th^ quintile is being taken. Authors need to highlight more studies that justify the reason behind choosing this distribution symmetry.

🡪 We thank the reviewer for this comment. In the revised manuscript, we have added a justification for selecting the 25th, 50th (median), and 75th quantiles. These three quantiles are widely used in the quantile regression literature because they capture the lower, central, and upper portions of the conditional distribution while maintaining distributional symmetry and avoiding sparsity at the extremes. We now cite Bilgili et al. (2021 who adopt the same quantile structure.

6. Spatial plotting, graphical representation can be given instead of clustered tables to make it more easily understandable.

🡪 For a better presentation of the results, the two largest tables have been relocated to the Appendix in the revised manuscript.

7. In order to ensure the precision of the results confidence interval needs to be given along- side the coefficient.

🡪 We thank the reviewer for this observation. Confidence intervals were not included because the quantile regression framework incorporates robust validation procedures, such as VIF diagnostics and Pseudo-R² measures. These provide reliable information about model precision and specification quality. Furthermore, confidence intervals in quantile regression are often broad and asymmetric, particularly when the dependent variable is a bounded synthetic indicator, making them difficult to interpret and providing little additional meaningful insight beyond the reported coefficients, robust standard errors, and significance levels. However, if required, confidence intervals can be added in an appendix.

8. Although, the idea of the study is noble and the objectives are interesting, and overall manuscript is fine but the authors need to put more emphasis on the policy implication. The way in which it can help in coping regional disparities in subjective well- being across Europe.

🡪 In the revised manuscript, the Conclusions section has been expanded to strengthen the discussion of policy implications. Now it clarifies how the synthetic SWB indicator and the quantile regression results can support strategies to reduce regional disparities in subjective well-being across Europe.

We want to thank the editor and reviewers for their helpful comments and suggestions. We believe that the revisions to this version address their concerns and that the paper is now ready for publication.
